# Circadian Regulation of Glutamate Transporters

**DOI:** 10.3389/fendo.2018.00340

**Published:** 2018-06-21

**Authors:** Donají Chi-Castañeda, Arturo Ortega

**Affiliations:** Laboratorio de Neurotoxicología, Departamento de Toxicología, Centro de Investigación y de Estudios Avanzados del Instituto Politécnico Nacional, Mexico City, Mexico

**Keywords:** circadian rhythms, clock genes, EAATs, glutamate transporters, neurodegenerative disorders

## Abstract

L-glutamate is the major excitatory amino acid in the mammalian central nervous system (CNS). This neurotransmitter is essential for higher brain functions such as learning, cognition and memory. A tight regulation of extra-synaptic glutamate levels is needed to prevent a neurotoxic insult. Glutamate removal from the synaptic cleft is carried out by a family of sodium-dependent high-affinity transporters, collectively known as excitatory amino acid transporters. Dysfunction of glutamate transporters is generally involved in acute neuronal injury and neurodegenerative diseases, so characterizing and understanding the mechanisms that lead to the development of these disorders is an important goal in the design of novel treatments for the neurodegenerative diseases. Increasing evidence indicates glutamate transporters are controlled by the circadian system in direct and indirect manners, so in this contribution we focus on the mechanisms of circadian regulation (transcriptional, translational, post-translational and post-transcriptional regulation) of glutamate transport in neuronal and glial cells, and their consequence in brain function.

## Circadian biological clock

Life has adapted to 24-h rhythms, better known as circadian rhythms (1). Consequently, a large number of organisms have circadian clocks that anticipate daytime and establish endogenous 24-h rhythms, which organize their physiology and behavior (2, 3). These endogenous rhythms are synchronized with the environment through external signals, the so-called *zeitgebers* (“time giver” in German), being the light the principal time cue (4).

Intracellularly, the mechanisms involved in circadian regulation are transcription-translation feedback loops of a group of genes denominated *clock genes* (5–7). In mammals, Brain muscle arnt-like 1 (BMAL1) and Circadian locomotor output cycles kaput (CLOCK) complexes control the periodic expression of *Cryptochrome 1* and *2* (*Cry1* and *2*), and *Period 1* and *2* (*Per1* and *2*), whose protein products inhibit BMAL1 and CLOCK, as well as their own transcription (5–8). These circadian transcription factors regulate thousands of clock-controlled genes, which orchestrate diverse physiological, metabolic and behavioral functions, resulting in a synchronized organism (3). Most tissues and cell types in the body possess a molecular clock (peripheral clocks) synchronized by the principal pacemaker located in the suprachiasmatic nucleus (SCN) of the anterior hypothalamus (2, 3, 9). Approximately, around 2–30% of each tissue's transcriptome is rhythmically synthesized (10, 11).

In mammals, the SCN receives direct photic input from photosensitive retinal ganglion cells via the retinohypothalamic tract (RTH) (12, 13). This tract mainly uses glutamate (Glu) as its neurotransmitter; however, pituitary adenylate cyclase-activating peptide (PACAP) and substance P are two peptide co-transmitters that also participate in retino-hypothalamic transmission (14–16). Interestingly, it has been shown that both of these co-transmitters regulate Glu neurotransmission, although the mechanism by which it is carried out remains unknown (15, 17–19). *In vivo* and *in vitro* studies have identified both metabotropic and ionotropic Glu receptors in the SCN (20–22), although it has been demonstrated that specific distribution and abundance of each Glu receptor subunit differs in this structure resulting in different effects of Glu on SCN neurons (21).

## Glutamate

Glutamate (Glu), the main excitatory neurotransmitter in the mammalian central nervous system (CNS), activates two subtypes of Glu receptors: ionotropic (iGluRs) and metabotropic (mGluRs) (23–25). The first group refers to a family of ligand-gated ion channels that have been classified by means of their pharmacological properties into: N-methyl-D-aspartate (NMDA), and α-amino-3-hydroxy-5-methyl-4-isoxazolepropionate (AMPA) and kainate (KA) receptors (24). The second subtype of Glu receptors belongs to class C of G-protein-coupled receptors, and its classification is based on the homology of their sequences, pharmacology, and signal transduction mechanisms (23, 25). It includes group I (mGluR1 and mGluR5), group II (mGluR2 and mGluR3) and group III (mGluR4, mGluR6, mGluR7, and mGluR8) (23, 25). Both subtypes of Glu receptors are widely expressed on pre- and post-synaptic terminals as well as on astrocytes that surround synapses (23, 26, 27).

Glu concentration in the synaptic cleft is in the low millimolar range (28, 29). However, after periods of intense glutamatergic activity, an excessive extracellular Glu concentration leads to an overstimulation of Glu receptors resulting in neuronal death, a phenomenon known as excitotoxicity, which is involved in neurodegenerative diseases (26, 30). In this context, Glu uptake from the extracellular space plays an essential role in the prevention of excitotoxic insults (28). A family of Na^+^-dependent high affinity Glu transporters carries out the Glu removal from the synaptic space. The excitatory amino acid transporters (EAATs) comprise five different Glu transporters: Glu/aspartate transporter (GLAST), Glu transporter 1 (GLT1), excitatory amino acid carrier 1 (EAAC1), excitatory amino acid transporter 4 (EAAT4), and excitatory amino acid transporter 5 (EAAT5) or EAAT 1-5 according to rodent and human nomenclature, respectively (28, 31–36). These transporters display a 50–60% amino acid sequence similarity, although different pharmacological and molecular properties, structure, and expression patterns are present for each subtype (28, 37). Within the CNS, Glu transporters have differential cell expression (glial or neuronal) (31, 36, 38–40). GLAST and GLT1 are found predominantly in the astrocytic plasma membrane (38–40), whereas EAAC1/EAAT4/EAAT5 are neuronal transporters mainly localized in hippocampal neurons, Purkinje cells, and rod photoreceptor and bipolar cells of the retina, respectively (31, 35, 36, 38, 41). However, GLT1 expression in neurons (28, 42–44), as well as EAAC1 and EAAT4 immunoreactivity in cortical and spinal cord astrocytes have also been reported (45, 46). GLAST and GLT1 carry out ~80–90% of the Glu uptake in the brain (28), and decreased expression and/or malfunction of these Glu transporters are related to several neurodegenerative disorders like Parkinson's, Huntington's and Alzheimer's diseases (47–49).

## General characteristics of glutamate transporters in neurodegenerative diseases

Through an antisense approach, it has been demonstrated that Glu transporters malfunction is involved in neurodegeneration in normal animals (47). Subsequently, Tanaka and colleagues reported, in mice lacking GLT1, a decrease of transport activity, lethal seizures and increased susceptibility to neurotoxicity (48). Years later, several research groups have demonstrated the role of Glu transporters in various neurodegenerative diseases. For example, Alzheimer's disease (AD) patients and animal models display a dramatic decrease in Glu transporters protein expression and in Glu uptake that is not correlated to its mRNA levels, demonstrating that other levels of regulation are present (50–54). In addition, Scott and coworkers described that GLT1 mRNA alternative splicing controls Glu uptake both in disease and in normal conditions (55). Moreover, glial Glu transporters have aberrant expression in distinct types of neurons (56, 57).

In the case of Parkinson's disease (PD), as with AD, there is also a decrease in Glu uptake; in PD, Glu transporters have an unusual trafficking between membrane and cytoplasm leading a decrease in Glu transporters at the plasma membrane (58). This phenomena relies in Glu transporters' ubiquitination by the E3 ubiquitin ligase Nedd4-2 (neuronal precursor cell expressed developmentally down-regulated 4–2) (58).

Likewise, Glu transporters have a critical role in Huntington's disease (HD), in which the expression of these transporters is diminished, the symptoms of HD worsen (59). In this sense, it has been demonstrated that aberrant huntingtin reduces GLT1 activity, either by dysfunction of the transporter itself or a transcriptional down-regulation, aggravating excitotoxicity (59, 60).

It is well-known that Glu transporters are regulated at different levels, at the transcriptional translational and post translational levels through modifications of transporter protein, as well as by the transporter targeting and trafficking (61–64). Nevertheless, there is compelling evidence demonstrating that Glu transporters are regulated in a circadian fashion.

## Circadian regulation of glutamate transporters

### Transcriptional, translational, and post-translational regulation

Until today, it has been demonstrated that in SCN both *Glast* mRNA and protein levels present a diurnal rhythm in 12/12 h light-dark conditions (65). According to these results, it has been proven that in the *Per2* mutant mice, GLAST protein is arrhythmic, highlighting the presence of a circadian regulation (65). Subsequently, using a cortical astrocytes culture from *Npas2* and *Clock* mutant mice, it was reported a decrease in *Glast* mRNA and protein levels, implying that glial Glu uptake is modulated via clock genes expression: *Per2, Clock*, and *Npas2* (66, 67). CLOCK and NPAS2 proteins are involved in *Glast* transcription or in *Glast* mRNA translation and/or stability (28), while PER2 modulates GLAST and by these means Glu uptake. In this sense, modifications in NPAS2 and/or CLOCK diminish PER2 levels and Glu uptake (66). More recently, it has been reported that glial Glu uptake within the SCN is modulated in a diurnal fashion (high levels of uptake during the light phase) but it does not exhibit circadian fluctuations (68). Leone and colleagues also report that Glu uptake activity does not change in constant darkness (68). It is important to mention that the possibility that Glu uptake is regulated by circadian clock *in vivo* cannot be ruled out. In line with these results, another research group also reported that Glu uptake in SCN is increased during the circadian day (22). Brancaccio and coworkers demonstrated that astrocytes modulate circadian timekeeping in SCN through glutamatergic signaling, and identified the presence of self-sustained circadian oscillations of Glu extracellular levels (22). The authors suggest that, in the light phase, Glu uptake is mediated by EAATs, including GLAST, GLT-1, and EAAC1 (22). These results could indicate that both Glu release and uptake are regulated in a circadian fashion.

It is reasonable to suggest that when there is a lack of GLAST transporter, compensation via upregulation of GLT1 is favored (65). For instance, in the *Per2* mutant mice it has been determined a shift in GLT1 protein maximal expression, from zeitgeber time 6 (ZT6, in control mice) to ZT18 (65), indicating that GLT1 protein is regulated by circadian clock. It is important to mention that shift in maximal expression of the GLT1 transporter correlates with ZT in which there is a downregulation of GLAST (65), suggesting that total uptake of Glu could be modulated by clock.

Through the use of *in situ* hybridization techniques in SCN, supraoptic nuclei, cingulate cortex and reticular thalamus of rats in constant darkness, it was found that *Eaac1* mRNA expression was rhythmic only in the SCN (69). Circadian expression of this transporter is associated with GABAergic activity regulation in the SCN, due an increased demand of GABA synthesis and release, immediately preceded by an increase in *Eaac1* mRNA expression (69). Increase in the expression of this transporter contributes to the neuronal clearance of Glu, which in fact is a precursor of GABA. Within the SCN, 95% of neurons are GABAergic (70), and together with astrocytes regulate circadian timekeeping through glutamatergic signaling (22), suggesting an important role of Glu transporters in the internal timekeeping system. In contrast, Kinoshita and colleagues could not find any a circadian-mediated *Eaac1* mRNA expression neither in serum-shocked SH-SY5Y cells and mouse mesencephalon by qRT-PCR (71). Taking together, these results suggest that temporal changes in *Eaac1* mRNA might be controlled by circadian clock in a tissue-dependent fashion. In addition, Kinoshita and collaborators also described that EAAC1 protein expression exhibits a diurnal variation in a 12/12 h light/dark cycle in mouse mesencephalon (71).

### Post-transcriptional regulation (circadian MicroRNAs)

In recent years, the proposal for a novel circadian regulatory system has been gaining ground. MicroRNAs (miRNAs) are a good example of a system that can rapidly respond to external stimuli since it is activated without changes in transcription and/or translation (71). In this context, miRNAs have revealed to be a key factor in the regulation of several circadian components (72–75). It has also been proved that peripheral oscillators exert circadian regulation over miRNAs expression (73–78). Increasing evidence indicates that miRNAs controlled by the circadian clock, regulate Glu transporters. Thus, miRNA-124 increases GLAST expression (79), while miRNA-142-3p and miRNA-155-5p decrease it (80, 81). Moreover, it has been demonstrated that miRNA-124 and miRNA-181a positively regulate GLT1 (82, 83), while miRNA-107 inhibits GLT1 expression (84). Specifically, EAAC1 rhythm is negatively controlled by miRNA-96-5p (71), miRNA-26a-5p (85) and miRNA-101b (86). This former miRNA also negatively regulates to EAAC1 protein (86). However, no evidence shows that miRNAs can target EAAT4 and EAAT5.

## Future directions

In the last two decades, several research groups have examined the different signaling pathways that modulate glial Glu transporters expression (GLAST and GLT1). Scarce information about EAAC1, EAAT4, and EAAT5 transporters is available. Particularly, EAAC1 has a much less evolutionarily conserved sequence in the 5′ noncoding region compared to GLAST and GLT-1, hindering the identification of *cis*- and *trans*-elements involved in its transcriptional regulation. Specifically, the circadian regulation of Glu transporters is an emerging theme that promises to be an indispensable tool in the preventing and/or treatment of diseases related to alterations in glutamatergic system. Future research should be directed to study of molecular mechanisms involved in circadian modulation of these transporters.

## Conclusion

**Figure 1:**
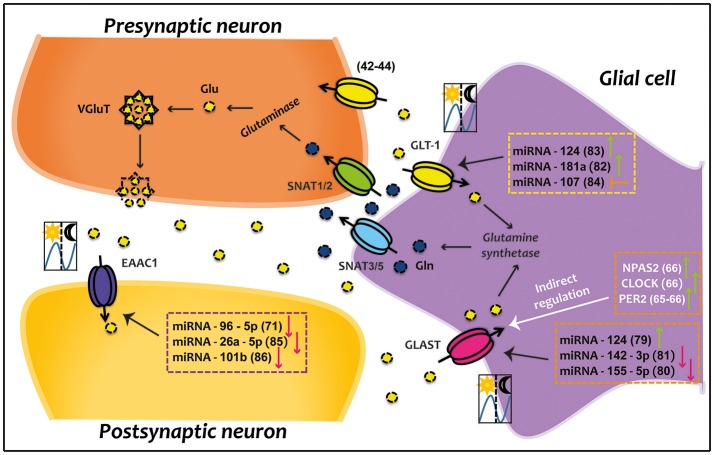
Direct and indirect circadian regulation of EAATs. Glutamatergic synapse which is composed of presynaptic neuron, postsynaptic neuron and glial cell compartment are represented. Some clock genes indirectly up-regulate GLAST; while several miRNAs directly down- or up-regulate GLAST, GLT-1, and EAAC1. Green arrows represent up-regulation, red arrows indicate down-regulation, and orange arrow denotes inhibition. The illustration of day/night indicates that transporter present a circadian rhythm in 12/12 h light/dark conditions. Numbers in parentheses refer to cited publications. CLOCK, circadian locomotor output cycles kaput; EAAC1, excitatory amino acid carrier 1; GLAST, glutamate aspartate transporter; Gln, glutamine; GLT-1, glutamate transporter 1; Glu, glutamate; NPAS2, neuronal PAS domain-containing protein 2; PER2, period 2; SNATs, sodium-coupled neutral amino acid transporters; VGluT, vesicular glutamate transporter.

Optimal functioning and precise regulation of Glu removal from the synaptic cleft is critical to prevent an excitotoxic insult and thus avoid several neurodegenerative pathologies. To date, compelling evidence suggests that Glu transporters could be regulated in a circadian fashion (Figure 1). It is clear that desynchronization or aberrant functioning of circadian system results in significant health consequences. In this sense, disruptions in the circadian regulation of Glu transporters is likely to be involved in neurological disorders like Parkinson, Huntington and Alzheimer diseases. Therefore, a better understanding of the molecular mechanisms that participate in the circadian regulation of EAATs might prove important for the proper development of therapeutic strategies aimed to prevent and/or treat pathologies related to excitotoxicity.

## Author contributions

DC-C gathered the relevant information, wrote the manuscript, as well as elaborated the figure. AO revised and edited the final version of the manuscript.

### Conflict of interest statement

The authors declare that the research was conducted in the absence of any commercial or financial relationships that could be construed as a potential conflict of interest.
